# Changes in Bacterial Gut Composition in Parkinson’s Disease and Their Metabolic Contribution to Disease Development: A Gut Community Reconstruction Approach

**DOI:** 10.3390/microorganisms12020325

**Published:** 2024-02-04

**Authors:** Johanna Forero-Rodríguez, Johannes Zimmermann, Jan Taubenheim, Natalia Arias-Rodríguez, Juan David Caicedo-Narvaez, Lena Best, Cindy V. Mendieta, Julieth López-Castiblanco, Laura Alejandra Gómez-Muñoz, Janneth Gonzalez-Santos, Humberto Arboleda, William Fernandez, Christoph Kaleta, Andrés Pinzón

**Affiliations:** 1 Bioinformatics and Systems Biology Research Group, Genetic Institute, Universidad Nacional de Colombia, Bogotá 111321, Colombia; ljforeror@unal.edu.co (J.F.-R.); jdcaicedon@unal.edu.co (J.D.C.-N.); julalopezcas@unal.edu.co (J.L.-C.); 2 Medical Systems Biology Research Group, Institute of Experimental Medicine, Christian-Albrechts-Universität zu Kiel, 24118 Kiel, Germanyj.taubenheim@iem.uni-kiel.de (J.T.);; 3 Neurosciences Research Group, Genetic Institute, Universidad Nacional de Colombia, Bogotá 111321, Colombia; 4 PhD Program in Clinical Epidemiology, Department of Clinical Epidemiology and Biostatistics, Faculty of Medicine, Pontificia Universidad Javeriana, Bogotá 110231, Colombia; mendieta-c@javeriana.edu.co; 5 Department of Nutrition and Biochemistry, Pontificia Universidad Javeriana, Bogotá 110231, Colombia; 6 Cell Death Research Group, Medical School and Genetic Institute, Universidad Nacional de Colombia, Bogotá 111321, Colombia; 7 Structural Biochemistry and Bioinformatics Laboratory, Pontificia Universidad Javeriana, Bogotá 110231, Colombia

**Keywords:** computational modeling, diet, gut microbiome, metabolic modeling, Parkinson’s diseases

## Abstract

Parkinson’s disease (PD) is a chronic and progressive neurodegenerative disease with the major symptoms comprising loss of movement coordination (motor dysfunction) and non-motor dysfunction, including gastrointestinal symptoms. Alterations in the gut microbiota composition have been reported in PD patients vs. controls. However, it is still unclear how these compositional changes contribute to disease etiology and progression. Furthermore, most of the available studies have focused on European, Asian, and North American cohorts, but the microbiomes of PD patients in Latin America have not been characterized. To address this problem, we obtained fecal samples from Colombian participants (*n* = 25 controls, *n* = 25 PD idiopathic cases) to characterize the taxonomical community changes during disease via 16S rRNA gene sequencing. An analysis of differential composition, diversity, and personalized computational modeling was carried out, given the fecal bacterial composition and diet of each participant. We found three metabolites that differed in dietary habits between PD patients and controls: carbohydrates, trans fatty acids, and potassium. We identified six genera that changed significantly in their relative abundance between PD patients and controls, belonging to the families *Lachnospiraceae*, *Lactobacillaceae*, *Verrucomicrobioaceae*, *Peptostreptococcaceae*, and *Streptococcaceae*. Furthermore, personalized metabolic modeling of the gut microbiome revealed changes in the predicted production of seven metabolites (Indole, tryptophan, fructose, phenylacetic acid, myristic acid, 3-Methyl-2-oxovaleric acid, and N-Acetylneuraminic acid). These metabolites are associated with the metabolism of aromatic amino acids and their consumption in the diet. Therefore, this research suggests that each individual’s diet and intestinal composition could affect host metabolism. Furthermore, these findings open the door to the study of microbiome–host interactions and allow us to contribute to personalized medicine.

## 1. Introduction

Parkinson’s disease (PD) is a progressive heterogeneous motor movement disease, primarily characterized by the loss of dopaminergic neurons in the substantia nigra pars compacta in the midbrain. These cells produce the neurotransmitter dopamine, which works in the striatum to modulate muscular tone and movement. These alterations lead to motor impairments such as tremors, rigidity, postural instability, and akinesia [1]. Although PD is known to primarily affect the basal ganglia, other nigral neurons, neurotransmitters and neuromodulators are altered; the latter include acetylcholine, glutamate, noradrenaline, and serotonin [2]. Moreover, the central and peripheral nervous systems are also affected, causing, in most cases, non-motor symptoms such as gastrointestinal alterations [3].

PD is the second most common neurodegenerative disorder after Alzheimer’s disease [4]. Globally, it has a prevalence of 100–300 per 100,000 people, and, in Colombia, PD prevalence is around 212.23 per 100,000 people [5]. Due to the increase in the elderly population worldwide, the number of people with PD is expected to double by 2030 [6]. Surprisingly, just 10% of the cases of PD are related to genetic background [7], and most cases are related to environmental factors that remain unknown. In search of the causes that promote it, additional environmental factors that could drive the development and/or progression of the disease have been suggested [8]. The gut microbiota has been postulated as one crucial environmental factor associated with PD-associated neurodegeneration, suggesting an important role in neural development and maintenance [9]. Thus, the microbiota and its modulators, such as the diet, have been suggested as therapeutic targets. Moreover, additional studies have also supported the hypothesis that diet impacts the development of neurodegenerative diseases such as PD [10].

Conversely, in PD patients, gastrointestinal symptoms often precede motor symptoms for years in the prodromal phase [3]. Braak et al. have suggested that some external pathological agents could be related to the disease’s etiology, by entering the host through either respiratory or gastrointestinal routes. According to this hypothesis, these agents would promote the development of PD [11], by inflammation, oxidative stress and alpha-synuclein misfolding in central and peripheral nervous systems [9].

To our knowledge, close to 20 cohort studies have described the composition of the PD gut microbiota compared with healthy controls [10,12,13,14,15,16,17,18,19,20,21,22,23,24,25,26,27,28]. Most of them have been carried out in North American, European, and Asian populations, but there are still no studies in the field from regions with different cultural and genetic backgrounds, such as Latin America. Earlier findings related to taxa associated with the disease are often inconsistent between different studies, maybe due to diverse causes of variability, such as genetic background or cultural and dietary differences. Therefore, to evaluate these differences as potential prognostic approaches and find adequate treatment targets, it is necessary to characterize the gut microbiome in diverse populations worldwide, including Latin American countries, where this type of information is lacking. Thus, this work aims to partially fill this gap, by characterizing a Latin American population microbiome in the context of PD, considering key aspects such as the diet and its effect on the gut microbiota community complexity.

To understand the complex interactions between environmental conditions and individual microbiome characteristics, a variety of approaches are needed. Meta-barcoding techniques, along with genome-scale metabolic modeling (GSM), hold promise. GSM offers advantages such as representing cell metabolism and generating plausible hypotheses that can be modeled for personalized medicine and research [29].

In GSM reconstruction and analysis, biochemical metabolic reaction flux rates can be predicted under specific input, and as a result, the personalized or general community microbiota can be simulated. Several gut bacterial reconstructions for humans have been previously reported and made available at the Assembly of Gut Organisms through Reconstruction and Analysis (AGORA) database [30], a public resource with growing importance in gut microbiome modeling. For instance, analysis toolboxes such as constraint-based reconstruction and analysis (COBRA) [31] using AGORA have been applied to study diverse diseases, including Parkinson’s disease (PD) [28], highlighting the importance of host–microbiome crosstalk in human health.

Through the characterization of a distinct microbiome from a group of the Colombian population, the present study aims to advance our understanding of the impact of the intestinal microbiota on the development of Parkinson’s disease. By characterizing a distinct microbiome from a group of the Colombian population, this pioneering PD-microbiome study in South America employed 16S sequencing and personalized computational modeling of the human microbiome, which integrates patient-specific microbial composition and diet. Notably, the detailed dietary information for each participant has been instrumental in identifying possible metabolic mechanisms by which the microbiome affects neurodegeneration. Moreover, the study has revealed potential changes in the predicted production metabolites associated with the metabolism of aromatic amino acids and the diet, suggesting that these factors may play a role in the development of the disease.

## 2. Materials and Methods

### 2.1. Study Subjects

Originally, 56 age (max ± 2 years difference) and sex-matched subjects (31 PD patients, 25 control subjects) were recruited to participate in the study. Six were excluded based on the inclusion/exclusion criteria, bringing the total number of subjects to 50 (25 PD patients, 25 control subjects); 22 male and 28 female. The study was approved by the ethics committee of the National University of Colombia and all participants gave informed consent.

The patients’ diagnosis was made by an experienced movement disorder specialist neurologist using the Webster Rating as an impairment scale for Parkinson’s disease, as well as data obtained from having access to their clinical histories. The exclusion criteria used were as follows: (1) regular use of probiotics or antibiotics during the last 3 months before sample collection, (2) secondary Parkinsonism, (3) familial Parkinson, (4) gastrointestinal primary diseases, (5) other neurological or psychiatric alterations, and (6) changes in dietary habits. For the controls, the exclusion criteria were as follows: (1) regular use of probiotics or antibiotics the last 3 months before the sample collection, (2) gastrointestinal primary diseases, (3) neurological or psychiatric alterations, and (4) changes in dietary habits.

### 2.2. Nutritional Data

A registered dietitian nutritionist developed a complete nutritional assessment adapted to the investigation’s purposes on all 50 subjects (25 cases and 25 controls) through a home visit from June 2018 to February 2019. This evaluation included a malnutrition screening test with the Ferguson screening tool [30], nutritional history, anthropometric evaluation, food pattern, and nutritional intake.

We evaluated the food intake with two 24 h recalls of the multiple steps, designed by the United States Department of Agriculture (USDA). The food pattern of the last six months was identified using a qualitative food frequency questionnaire, modified according to the National Survey of Nutritional Situation 2005 and 2010 [31], the Dietary Guidelines for Americans, and risk tracer nutrients concerning microbiota composition and Parkinson’s disease. Every food was coded and analyzed using the Colombian Food Composition Table—2015 [32] and the Food Composition Lists of the USDA [33], Finland [34], and Germany [35], comprising 60 nutrients. Additionally, we evaluated the food frequency questionnaire according to the periodicity on a 10-point scale endorsed by USDA [36].

In the anthropometric assessment, weight, height, or height estimation by knee heel height with the equation developed for Chumlea [37] was used. We assessed the muscle mass using the arm and calf circumferences and fat mass using the triceps skinfold circumference. The analysis added BMI stratification by the cut-off point for adults [38] and older adults [39]. The estimation of muscle and fat mass was measured according to the cut-off points established in the Third National Health and Nutrition Examination Survey (NHANES) [40] and Frisancho [41] for arm muscle circumference.

Additionally, we contrasted the intake of nutrients following the established dietary recommendations of the Colombian Resolution 3803 of 2016 [42], “Recomendaciones de Ingesta de Energía y Nutrientes (RIEN)”, which is the adaptation of the Dietary Reference Intakes (DRI) [43]. The intake value of each nutrient was compared against the following values: adequate macronutrient distribution range (AMDR), estimated average requirement (EAR), recommended dietary allowances (RDA), adequate intake (AI), or tolerable upper intake level (UL), to establish whether the intake of each nutrient was in deficit or exceeded the recommended value. Only those nutrients reported in the recommendations were taken for analysis.

### 2.3. Sample Collection and DNA Extraction

Fecal samples were obtained by all 50 participants. Samples were collected at home, following physician indications, into sterile disposable containers. The fecal samples were collected in the morning hours, frozen, and stored at −20 °C for further processing (maximum 3 days later). The ZymoBIOMICS DNA Miniprep Kit (Zymo Research, Irvine, CA, USA) was used according to the manufacturer’s instructions and the in-house DNA extraction protocol refinement. After extraction (three technical replicates per sample), DNA samples were quantified using Nanodrop ND-1000 (Thermo Fisher Scientific, Wilmington, DE, USA) and Qubit Invitrogen (Life Technologies, Carlsbad, CA, USA), and the integrity of the DNA was evaluated by 0.8% agarose gel electrophoresis.

### 2.4. 16S rRNA Gene Amplicon and Sequencing

The DNA extracts were transferred to 96-well plates. Library preparation and sequencing were performed by The University of Iowa (United States) sequencing services (Iowa State University DNA Facility). The hypervariable regions of the bacterial 16S rRNA gene V4-V5 were amplified using the universal 16S forward primer (515F: GTGYCAGCMGCCGCGGTAA) and the reverse primer (926R:CCG CAA TTTTTTTT). The DNA libraries were then multiplexed for sequencing on the Illumina MiSeq platform (2 × 250 paired-ends) in a single flow cell lane.

### 2.5. Bioinformatics and Statistical Analysis

The already demultiplexed data were obtained. All raw sequence data generated in this study have been deposited in the National Center for Biotechnology Information NCBI under BioProject accession number PRJNA975118. The replicates in each sample were concatenated and subsequently filtered using prinseq-lite-0.20.4 program [44]. Bases below Q-score 24 were cut off at the start and the end of the reads. After processing, the unique sequences and abundances were obtained, the chimeras were filtered, and biological sequences were predicted into zero-radius operational taxonomic units (zOTUs) using the unoise3 algorithm part of the Usearch program [45,46]. Sequences with less than 400 bp were removed from the raw data. Taxonomic assignments were obtained using the Ribosomal Database Project RDP training set v16 at an 80% confidence level. 

Since the highest taxonomic level was the genus, the specific species of *Streptococcus* were identified using a direct BLAST analysis between zOTU31 and zOTU59 and the NCBI’s nucleotide database. BLAST analysis indicates that zOTU31 shares a 100% identity with the *Streptococcus thermophilus* strain TMPC 45524 16S ribosomal RNA gene, partial sequence (ID: ON358420.1).

Statistical assessment and data visualization were performed using R (version 4.1.0), code available in Github repository: https://github.com/ljforeror/Parkinson_Microbiome (accessed on 1 December 2023). To consider significant comparisons, *p*-values < 0.05, or adjusted *p*-values < 0.05 were taken into account. To evaluate potentially confounding clinical variables, we used either Student’s *t*-test, the Wilcoxon signed rank test, or Fisher’s exact test, depending on the type and distribution of each variable. Data were plotted by means of ggplot2 (v. 2_3.3.5).

The structure and composition analysis of the bacterial community was performed using the R package phyloseq [47] (v. 1.36.0). The library size of each sample was normalized using rarefying to even sequence depth and exclusion taxa present in less than 20% of all samples. Bacterial diversity and richness were also analyzed using phyloseq with alpha diversity indices (observed richness, Shannon index, and inverse Simpson index). A Wilcoxon rank-sum test was used to evaluate alpha diversity differences.

To evaluate beta diversity, phyloseq (v. 1.36.0) was also used with Bray–Curtis dissimilarity. Distance measures, as an unweighted unique fraction metric and weighted Unifrac, were used for unconstrained ordination on genus proportions between the PD and control groups. The command ANOSIM (analysis of similarities) and Adonis (permutational multivariate analysis of variance using distance matrices) were run, with the parameter perm = 9999, implemented in vegan (v. 2.5–7) [48]. Data were plotted using non-metric multidimensional scaling (NMDS) in the R package ggplot2 (v. 2_3.3.5).

We evaluated the differential abundance between the PD and control groups by using the test for differential expression based on a model using the negative binomial distribution of DESeq2 (v. 1.32.0), by means of the phyloseq package. Comparisons on three taxonomic levels (zOTU, genus, and family) between PD and control groups were performed.

#### Clinical and Diet Correlations in Healthy and Parkinson’s Disease Bacterial Compositions

Based on the demographic, anthropometric, and clinical information recorded for both PD patients and controls, as well as the nutrient quantification performed as described above, a correlation analysis was performed using RStudio. For this purpose, the data containing clinical, anthropometric, demographic, and clinical information (age, weight, alcohol or tobacco consumption, number of stools per week, Bristol scale, disease duration, Webster scale, severity of phenotype Parkinson’s, and calf perimeter) and the number of uptake nutrients from the diet in patients and controls were used. We obtained the correlation values and *p*-values for the correlation coefficients. The *p* values obtained were corrected by the false discovery rate FDR method. The significant correlation between the clinical, anthropometric, demographic, and clinical variables and the nutrient values were taken with a significance *p*-value of <0.5. Finally, for Spearman’s correlation between dietary information and the most representative zOTU, we followed the mentioned steps.

### 2.6. Individualized Microbial Community Reconstructions

To predict the functional composition of the gut microbiome from 16S data, we used PICRUSt (v.2-2.3.0-6). To predict metabolic processes in the microbial community, we selected the AGORA collection (v.1.02) models, based on the individual composition and abundance found in the participants in our cohort. The AGORA collection comprises genome-scale metabolic models of 818 common human gut microbial species.

Thus, we took the 16S rRNA gene-filtered sequences belonging to the zOTUs obtained and mapped them to their corresponding models contained in the AGORA collection, using the MicrobiomeAGORA package https://github.com/mucosimmunol/aTNF-AZA/tree/master/microbial_community_modellings (accessed on 13 May 2023) [49]. We required a minimum sequence identity of 97% and thus found the closest neighbor based on 16S sequence information. We also took the diet information for each individual, obtained the mM (milliMolar) equivalent value [50] for all nutrients, and set it as the input in each of the personalized *in silico* microbiota, by constraining the lower bounds of the inflow reactions of the corresponding metabolites.

In order to model the community behavior of each of the previously identified microbiome compositions, we used the R package BacArena v.1.8 [51]. In BacArena, bacteria are represented by individual genome-scale models, which are placed in grids. Inside each grid, the models can move randomly, can take and exchange metabolites that are produced or consumed by other bacteria, or can just accumulate in the environment. We set the size of the environment to 3600 grid cells where the microbe models can grow with enough room to reduce the stationary phase. There were 300–500 microbes initially included based on their relative abundances, as evidenced by previously described bioinformatics 16S analysis. The simulation was made for each individual sampled, including input components extracted from 99% diet data of each participant and 1% of Western diet reported in Virtual Metabolic Human VHM (to satisfy some model-specific growth requirements). Each metabolite obtained from the diet was converted to grams, then the weight was divided by molecular weight to obtain mol/L. Finally, they were multiplied by 1000 to obtain the values in mmol/L. All simulations were performed using ten replicates in parallel and 12 h of bacterial growth. In these individual-specific bacterial gut community models, we obtained the concentrations (amount of the substances in mM) of metabolic end products at the end of the simulation. Significant differences between healthy controls and PD patients were determined using Wilcoxon rank sum tests and generalized linear modeling.

### 2.7. Software

The simulations and their analysis were performed in the R environment (v.4.1.0 and v.3.6.1), using the BacArena [52] and sybil (v.2.2.0) packages [53]. The following additional packages were used: sybilSBML (v.3.1.2), linear programming solver CPLEX (v. 12.7.1), and the R package cplexAPI (v.1.4.0). For parallel computing, the software parallel, with the foreach package (v.1.5.2), and doParallel (v.1.0.17) were used.

## 3. Results

### 3.1. Clinical and Demographic Characteristics of the Studied Population

The clinical and demographic characteristics of the participants in the study were compared between groups. They showed significant differences in variables, such as Bristol stool scales, pramipexole medication, calf perimeter, and alcohol habits. The increased calf perimeter in controls compared to PD patients indicates that PD patients have a loss of muscle mass [41]. PD participants took anti-Parkinson medication, and they did not have special diets or infectious diseases. Some participants had pre-diabetes, but just two had metformin prescribed as treatment (Table 1).

### 3.2. Nutritional Analysis

#### 3.2.1. Dietary Requirements

Fifty percent of the participants presented with an inadequate consumption of protein, 31% presented with an inadequate consumption of fat, and 27% of carbohydrates. Within their nutritional diagnosis, it is noteworthy that 35% of the participants presented with excess weight, 23% with nutritional risk, and 42% with some degree of malnutrition (Supplementary Tables S1 and S2).

#### 3.2.2. Clinical Correlations in Parkinson’s Disease and Dietary Metabolites

**Figure 1:**
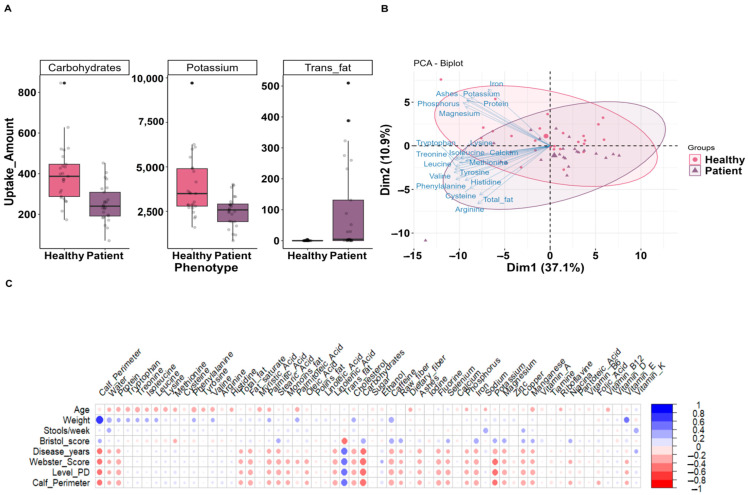
Dietary metabolites consumption between PD patients and controls: (**A**) box plots for the dietary metabolites consumed for the two study groups. Differences across the two study groups are significant (*p* < 0.05); (**B**) principal components analysis of dietary metabolites consumed by 50 participants dot and triangle indicates the phenotype (Healthy and Patient), ellipse colors purple and pink represent the phenotype, and a larger size of dot and triangle represents the centroid; and (**C**) heatmap representing the correlation analysis performed between clinical variables and nutritional data.

To provide an additional indication about the dietary contributors to clinical characteristics related to an increased risk of PD, a correlation analysis was performed between 10 clinical, anthropometric, and demographic variables and 65 quantified nutrients in samples from our cohort of PD patients and controls. We obtained 11 significant correlation coefficient (rs) values of nutrients against one or more clinical variables. Interestingly, the level of carbohydrates presented a negative correlation with three clinical variables: disease duration (rs = −0.64), Webster’s scale (the quantification of motor manifestations) [54] (rs = −0.62), and PD severity scale (rs = −0.63). In addition, we found that the uptake of trans fatty acids (defined as the products of the hydrogenation of unsaturated oils or biohydrogenation in the stomach of ruminant animals, which increase the ratio of low-density lipoprotein LDL cholesterol) [55] showed a positive correlation with the Webster score (rs = 0.62) and with the PD severity (rs = 0.56) (Figure 1C, Supplementary Table S3). Based on the non-normality assumption, a Wilcoxon test was used to compare the dietary intake between the two groups (patients and controls), as shown in Table 2. We found that PD patients have a significantly increased intake of trans fatty acids, and a significantly decreased intake of carbohydrates and potassium (Figure 1, Supplementary Tables S5 and S6). This was in concordance with the variables cluster found in the principal component analysis (PCA) in each group studied (Figure 1B).

#### 3.2.3. Principal Component Analysis of Dietary Uptake

After analyzing the dietary data by means of principal component analysis (PCA), we found that the first principal component (PC1) is determined by the number of amino acids, protein, and some minerals in the diet (Figure 1B). PC2 corresponds to a diet rich in carbohydrates, caffeine, some vitamins, some fatty acids, and a few minerals. Finally, PC3 is represented mainly by trans fatty acids, vitamin B, ascorbic acid, thiamine, cholesterol, and a few vitamins and fatty acids. Hereby, dimension 1 seems to explain the variability shown on the PCA better than the other two dimensions. The group of metabolites close to potassium, magnesium, caffeine, and others represented in the biplot by close vectors seem to be characteristic of the healthy group, while the patients seem to be represented by trans fatty acids and amino acids (Figure 1, Supplementary Figures S2 and S3).

### 3.3. Species Richness, Alpha Diversity, and Beta Diversity

From three replicates of 50 fecal samples, including healthy and patient samples, and after filtering, we obtained an average of 1,401,450 reads per sample, and the library size was rarefied to 41,007 reads per sample (90% of sample abundance with fewer reads) by random subtraction (Supplementary Figure S4). There were 932 zOTUs, 180 genera, 75 families, and 11 phyla obtained from the 16S rRNA gene amplicon data. The top phylum abundances were similar between PD patients and healthy controls (Supplementary Figure S5).

**Figure 2:**
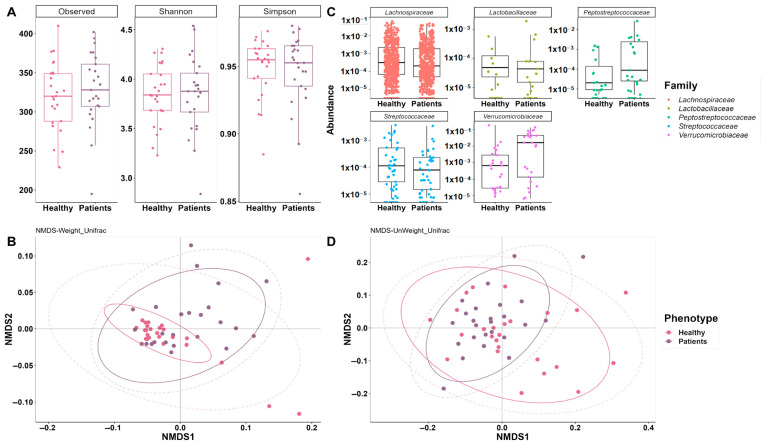
Alpha diversity and beta diversity differential abundance analysis of gut microbiota between PD patients and controls: (**A**) estimators of α diversity, observed species, Shannon, and Simpson indices in patients with PD and healthy controls. There are no significant differences between the groups; (**C**) abundance of families (each point represents the abundance of every zOTUs associated); (**B**,**D**) The composition of the samples was compared using the UniFrac distances with the normalized abundances. Weighted Unifrac considers the abundance of the species, whereas unweighted Unifrac only considers the information regarding the presence and absence of species.

We analyzed within-community microbial diversity using α-diversity analysis. We calculated observed-species, Shannon, and Simpson indices but did not find any difference between controls and patients (*p*-value < 0.5) by Wilcoxon rank-sum test (Figure 2A). Hence, there is no general difference in diversity between PD patients and healthy individuals, hinting at differences in the composition of microbiomes which are associated with PD.

Hence, we evaluated beta diversity to identify compositional differences between the two populations using Bray–Curtis, weighted, and unweighted Unifrac (Figure 2B,D, Supplementary Figure S6). We observed significant dissimilarities between patients and healthy controls based on weighted and unweighted Unifrac, by analysis of similarities (Anosim) and permutational multivariate analysis of variance (Adonis); despite this, the effect size (R value) was still small and the explained variability was ~2–8% only (Table 2, Supplementary Table S7).

### 3.4. Differential Abundance Analysis of Gut Microbiota between Parkinson’s Disease Patients and Controls

We evaluated the differential abundance between the PD and control groups by means of DEseq2 [56]. The differential abundance testing method is based on a model using the negative binomial distribution on taxonomic levels (zOTUs, genus, family, and phylum). This analysis suggested that some taxa were differing significantly between PD patients and controls (one phylum, two families, and six zOTUs) (Supplementary Tables S7–S10), with more than 0.1% relative abundance. In the differential abundance analysis, the zOTUs that were significantly decreased in PD were zOTU75 (genus: *Lactobacillus*) and zOTU31 (genus: *Streptococcus*). The zOTUs significantly increased in PD were as follows: zOTU11 (genus: *Akkermansia*); zOTUs35 (genus: *Clostridium Cluster XlVa*); zOTU59 (genus: *Streptococcus*); and zOTU66 (genus: *Intestinibacter*).

### 3.5. Inferred Functional Microbiota Profiling

**Figure 3:**
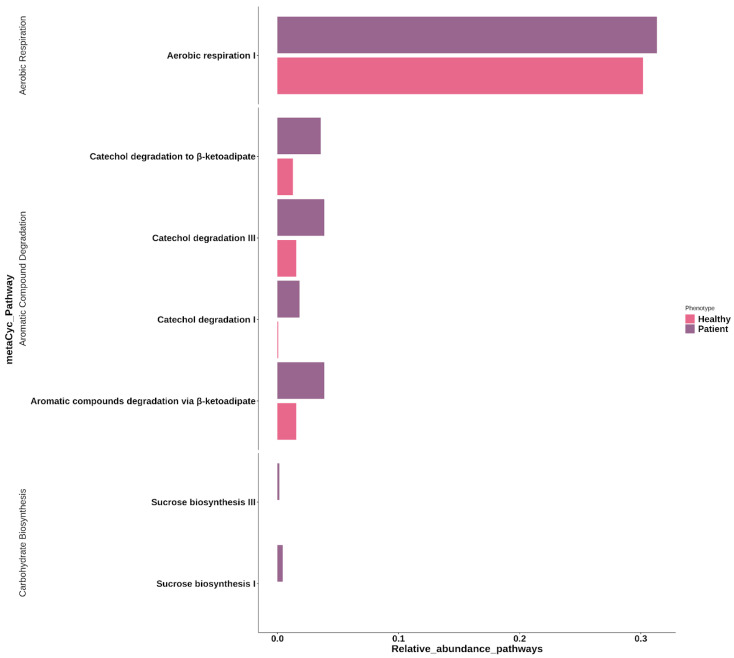
Bar plot representing the significant differential metabolic pathways in PD patients and controls: aerobic respiration I, catechol degradation to β-ketoadipate, catechol degradation III, catechol degradation I, aromatic compounds degradation via β-ketoadipate, sucrose biosynthesis III, and sucrose biosynthesis I pathways, from the bacterial information in each participant belonging to each group—PD patients (purple) and controls (pink).

In order to predict the functional alterations of the microbes in feces, PICRUSt2 [57] was applied to estimate the metaCyc pathway abundances, using the 16s rRNA gene sequencing data in PD and healthy groups. To identify functional abundance with significant differences between PD and healthy groups, DESeq2 [56] was used. We identified differences in aerobic respiration I (cytochrome c) (PWY-3781), sucrose biosynthesis I (from photosynthesis) (SUCSYN-PWY), sucrose biosynthesis III (PWY-7347), catechol degradation III (ortho-cleavage pathway) (PWY-5417), aromatic compounds degradation via β-ketoadipate (PWY-5431), catechol degradation I (PWY-5415); and catechol degradation to beta-ketoadipate (CATECHOL-ORTHO-CLEAVAGE-PWY) (Figure 3 and Supplementary Table S12); all of the pathways were found to be more abundant in the PD microbiome.

### 3.6. Microbiota Modeling

We aimed to gain a deeper functional understanding of the differences in microbiome metabolism between patients and controls and identify which microbial metabolites may be altered in PD, due to changes in the microbial community structure. We predicted the secreted metabolic end products of 50 simulated personalized *in silico* microbiota communities, using personalized diets from each participant to better represent individual biological conditions (Supplementary Table S13). The microbial communities were simulated using the individual-based modeling framework BacArena, which combines FBA (flux balance analysis) and individual modeling of the bacteria to study microbial metabolism and complex dynamics between interacting organisms [51]. Thus, the simulation can predict interactions and the production of metabolites, to identify signals of metabolic differences between PD and control phenotypes.

#### 3.6.1. Microbial Differences between Healthy Controls and PD Patients

The 16S rRNA sequence from each of the participants was mapped to AGORA. A total of 272 microbial strains from this resource were present in our participants. From these, we took the most abundant (with a relative abundance larger than 2%) microorganisms in each individual microbiota to be included in the simulation (147 microbial strains); thus, for our simulations, the microbiota with the smallest number of reconstructions contained 16 models and the largest had 52 models out of the total 147. Considering the overall abundance of species from the 16S data, on average, 64.1% of reads matched with species from the AGORA resource. Some genera included in the models were different compared with the 16S data (strains’ metabolic reconstructions). This can be explained by the patients and controls potentially having more diverse microbes than those reported in the database, or perhaps the resolution of the 16S regions does not allow the identification of the corresponding strains, which can lead to a lower abundance or different genera present in the simulation of each individual.

Performed simulations suggest that microorganisms can co-exist stably as a community, based on the *in silico* simulation in BacArena. Organisms included at the beginning of the simulation remained present after 12 h of growth. At the end of the simulation, the grid was populated with an average of 3534.95 (12 h) microbes, with a percentage of grid occupation of 98%.

#### 3.6.2. Extracellular Metabolites Concentration at 12 h Simulation between Healthy Controls and Parkinson’s Disease Patients

**Figure 4:**
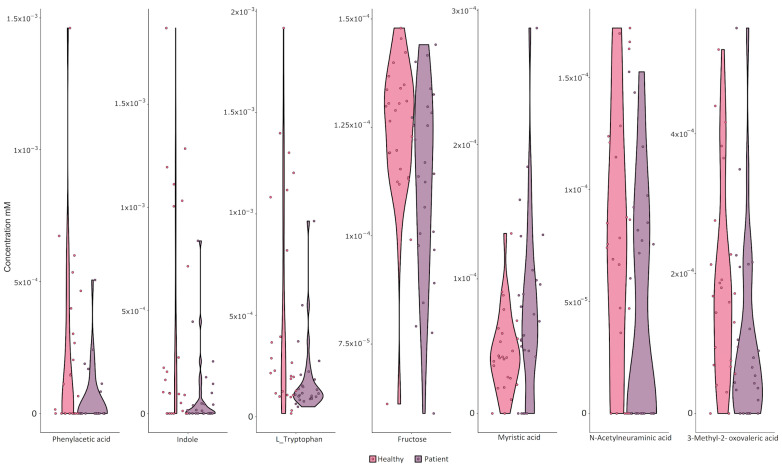
Violin plot of the significant differential metabolic metabolites between PD patients and controls simulations: phenylacetic acid concentration in mM at 12 h simulation in each participant belonging to each group of PD patients (purple) and controls (pink). Indole concentration in mM at 12 h simulation in each participant belonging to each group, PD patients (purple) and controls (pink). L-Tryptophan concentration in mM at 12 h simulation in each participant belonging to each group PD, patients (purple) and controls (pink). Fructose concentration in mM at 12 h simulation in each participant belonging to each group of PD patients (purple) and controls (pink). Myristic acid concentration in mM at 12 h simulation in each participant belonging to each group of PD patients (purple) and controls (pink). 3-Methyl-2-oxovaleric acid concentration in mM at 12 h simulation in each participant belonging to each group of PD patients (purple) and controls (pink). N-Acetylneuraminic acid concentration in mM at 12 h simulation in each participant belonging to each group of PD patients (purple) and controls (pink).

Based on simulation results, we evaluated whether there are metabolic differences between healthy and PD patients using different statistical tests. We compared the concentration of the final metabolites using generalized linear models (GLM) and the Wilcoxon test (Supplementary Tables S14 and S15). We were able to identify 134 common metabolites across all simulations. Based on this metabolite set, we observed the following seven significantly increased metabolites and one decreased metabolite in the healthy group before multiple testing corrections were carried out: phenylacetic acid (pac), indole (indole), L-Tryptophan (trp_L), D-Fructose (fru), myristic acid (ttdca), 3-Methyl-2-oxovaleric acid (3mop), N-Acetylneuraminic acid (acnam) (Figure 4 and Supplementary Table S14). We could not identify significant changes in the predicted metabolite concentrations after multiple testing corrections were carried out (Supplementary Table S15).

Each of the metabolites identified as differentially produced in the simulation are present in each model at different concentrations. Indole, tryptophan, fructose, phenylacetic acid, 3-Methyl-2-oxovaleric acid and N-Acetylneuraminic acid were found to be higher in the healthy phenotype simulation, while myristic acid was found to be higher in the PD phenotype simulation (Figure 4). Apart from these metabolites, common short-chain fatty acids SCFAs were identified without significant differences. The median acetate concentration was higher in the healthy models than in the PD models. The median butyrate concentration was higher in the PD models. Finally, propionate concentrations were higher in the healthy models (Supplementary Figure S6).

#### 3.6.3. Bacterial Reconstructions Producing or Consuming the Differential Metabolites in the Simulation between the Patient and Control Phenotypes

**Figure 5:**
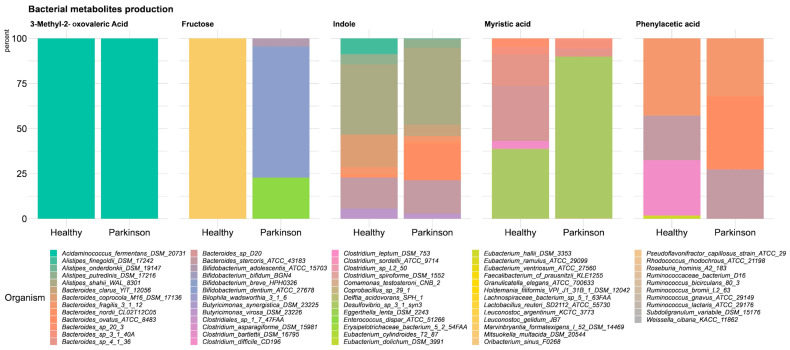
Differential metabolite-producing bacteria *in silico* between phenotypes: the largest producers of 3-Methyl-2-oxovaleric acid, myristic acid, indole, fructose, and phenylacetic acid. The percentage of production for each organism is indicated for the first major producers.

Since we had the exchange fluxes of each metabolite that is associated with PD, we identified which microorganisms are related to their consumption or production (Supplementary Tables S16 and S17). The values of the uptake and secretion fluxes (consumption and production) of each microbial reconstruction for each metabolite were assessed on each phenotype (healthy or PD) (Figure 5, Supplementary Figures S8–S10). We found that *Bacteroides* and *Desulfovibrio* were largely responsible for the production of myristic acid. Likewise, *Bacteroides* are involved in the production of indole and phenylacetic acid. Bacteria of the *Bifidobacterium* genus were producers, although of low amounts, of fructose, but the biggest amount of fructose is uptaken from many genus of *Acidaminococcus*, *Akkermansia*, *Alistipes*, *Bacteroides*, and others (Figure 5, Supplementary Figure S8).

## 4. Discussion

PD is a progressive heterogeneous motor movement disorder, characterized by the loss of dopaminergic neurons in the substantia nigra in the striatum. These alterations lead to motor impairments [1]. The gastrointestinal tract and the brain share a bidirectional signaling route, called the microbiota–gut–brain axis. With the advancement of high-throughput sequencing methods, the relationship between the gut microbiota and its influence on human physiology has been explored extensively. More recently, the gut microbiota has also been shown to have an integral role in neurodegeneration [58].

Several studies have characterized the gut bacterial composition in PD patients and healthy controls [10,12,13,14,15,16,17,18,19,20,21,22,23,24,25,26,27,28]. Here, we present the first characterization of the microbiomes of Parkinson patients from the Latin American population, which have been neglected in previous microbiome studies about microbial composition and its influence on disease development.

To address this gap, fecal matter was obtained from Colombian participants. Analysis of differential composition, diversity, and personalized computational modeling was carried out, given the individualized diet and fecal bacterial composition of the participants. We found three dietary metabolites and six genera that significantly changed in their relative abundances between PD patients and healthy controls, belonging to the families of *Lachnospiraceae*, *Peptostreptococcaceae*, *Verrucomicrobioaceae*, *Lactobacillaceae*, and *Streptococcaceae*. The personalized metabolic modeling of gut microbiomes revealed an increased concentration of seven metabolites, suggesting that the diet and the intestinal bacterial composition of each participant could affect the metabolism of the host and, thus, influence the development of the disease.

### 4.1. Six Zero-Radius Operational Taxonomic Units Changed Significantly in Their Relative Abundances between Parkinson’s Disease Patients and Controls

In our study, we found six significant differences between PD patients and healthy controls at the zOTU level, with a relative abundance over 0.1%. One of those zOTUs corresponds to the family *Verrucomicrobioaceae*, genus *Akkermansia* (zOTU11), and was increased in PD patients. This is an organism that has been described as differentially abundant also in recent studies of Parkinson’s disease. *Akkermansia muciniphila* has frequently been observed to increase in abundance in PD patients and in levodopa-naive PD patients [10,12,13,14,17,24,26,28,59,60].

This bacteria is a dominant human gut mucus colonizer that can produce mucin-degrading enzymes and uses the mucin as a carbon and nitrogen source [61]. Through the fermentation of mucin, it can produce sulfate and acids like acetic and propionic acids [62]. It has also been shown that, in the absence of dietary fiber, the abundance of *A. muciniphila* increases and could promote pathogen susceptibility [63]. It has been suggested that changes in intestinal mucin composition might facilitate the access of antigens and pathogens and increase inflammation [64]. Although we were not able to find any significant difference between groups in predicting functional abundances (metabolic pathways abundances using PICRUSt2) related to sulfur metabolism, this organism has been previously shown to have a role in sulfur metabolism identified before in PD patients [65]. Sulfur derivatives could, under conditions of dysbiosis, have detrimental effects on the colon, in addition to increased gut permeability, immune stimulation, and altered metabolism of SCFAs, and could also promote inflammation [28]. However, the explicit role of *A. muciniphila* in the context of PD remains to be elucidated. Thus, it has been suggested that it is important to explore diverse strain effects [59], and how diverse factors like medication [14] and diet [66] can be related to the function of this bacteria within the microbial community, in the gut and the context of PD.

Several studies of PD have reported a decrease in bacterial families like *Ruminococcaceae* and *Lachnospiraceae*, which include the genus *Faecalibacterium* and *Roseburia*. These bacterial families are butyrate producers [67]. This study identified an increased abundance of the family *Lachnospiraceae*, genus *Clostridium* Cluster XlVa (zOTUs35) in the PD group. It is a producer of butyrate in mucous membranes [68]. Butyrate is one of the most common SCFAs produced by gut bacteria, is related to different biological pathways, including energy homeostasis, oxidative stress, neural signaling, anti-inflammatory effects, glucose and lipid metabolism, and epigenetic modulation [69,70]. In mice susceptible models, butyrate can induce PD phenotype [58]. Nevertheless, the observation of the increased prevalence of the *Clostridium* Cluster XlVa genus in the PD population could be attributed to non-related medical conditions not taken into account during other studies, such as host genotype, variation among bacterial strains, diet, and lifestyle [71]. In the latter case, for instance, it has been shown that *Clostridium* Cluster XlVa abundance is elevated in patients suffering from depression [72]. It is therefore possible that our results for *Clostridium XIVa* could be a result of some other unknown condition. Thus, more research and characterization of gut dysbiosis and its role in various disease conditions are necessary.

Variations in etiological factors and the choice of analysis can translate into different outcomes when applied to data with underlying multi-variable dependencies involving patients and diseases from different populations. For instance, a decreased abundance of *Lactobacillus* of the family *Lactobacillaceae* (zOTU75) in PD patients was observed in our study. This is consistent with a study performed on Chinese PD patients [20,24] and one study with a cohort from Finland [17]. But the opposite was found in other PD studies [14,16,18,19,21,23,28,73]. In PD, one of the reasons that causes an increased abundance of *Lactobacillus* is the use of the drug levodopa [60]. The capacity of many *Lactobacillus* species to metabolize catecholamines may impact levodopa treatment, and the medication promotes its proliferation [74].

In our study, we observed a reduction in *Lactobacillus*, even though most patients in the cohort (18 patients) were under levodopa–carbidopa treatment. In one study, it was suggested that the increase in *Lactobacillus* in healthy controls could correlate with genetic, regional, and dietary differences, like the consumption of a diet enriched with *Lactobacillus* [17,20,24,75]. However, in our study, we did not observe the consumption of probiotics within the cohort. A possible explanation for the reduced levels of *Lactobacillus* could be associated with the relationship of bacteria of this genus with the regulation of genes involved in the tight junctions. Therefore, this could compromise the permeability of the barrier, which has also been associated with PD [24,76].

We also found an increased abundance of zOTU59 and a decreased abundance of zOTU31, both belonging to the genus *Streptococcus* from the family *Streptococcaceae*. This genus is a putative pathobiont that increases in PD [22,27,28]. It is reported that cadaverine is increased in PD patients and associated with Lewy body (LB) formation [77]. Like other polyamines (putrescine and spermidine), it has toxic effects in mice, promoting motility dysfunction and inflammation [1,78,79].

*Streptococcus thermophilus* is a Gram-positive bacteria that has been reported as a probiotic due to its beneficial effects in the host [80]. Additionally, it was shown that the water-soluble exopolysaccharide (EPS-1) has protective effects on acute mouse colitis by mitigating colonic epithelial cell injury, alleviating intestinal inflammation, and improving the mucosal barrier function [81]. In this regard, the decreased abundance of that zOTU in the PD population may be related to negative effects on the barrier permeability of the colon in PD patients.

With respect to zOTU59, we were able to find a 100% identity with the *Streptococcus lutetiensis* strain 2709 16S ribosomal RNA gene, partial sequence (ID: MT611722.1). *S. lutetiensis* is part of the *Streptococcus bovis*/*Streptococcus equinus* complex (SBSEC), a non-enterococcal group in the *D Streptococcus* spp. complex [82]. Although they have been described as strains that are safe and part of the diet [82], *S. lutetiensis* strains have also been reported in children with diarrhea in China. The genome annotation of *S. lutetiensis* has revealed the presence of pathogenic islands and virulence genes such as sortase (srtA), associated with adhesion and host colonization [83,84]. Therefore, the decreased abundances of zOTU31 and increased abundancies of zOTU59 in our study are confounding, and the exact role of these organisms in PD remains to be elucidated.

Finally, regarding zOTU66, we found *Intestinibacter* of the family *Peptostreptococcaceae* to be increased in PD patients, contrary to what has been previously reported [58,85]. Moreover, in a study on major depressive disorder and sleep quality, *Coprococcus* and *Intestinibacter* were shown to be associated with sleep quality, independent of the severity of depression [86]. The increased abundances can also be explained by differences in genetic makeup [87], geography [85], diet, and other diseases and/or disorders that could alter gut microbial composition [86]. In a study with a population from 50 to 80 years old, it was found that *Peptostreptococcaceae* abundance increased with the decreasing quality of life. Thus, its abundance could be detrimental to health [88]. Additionally, its increase has also been found in diverse diseases, such as type 2 diabetes mellitus [89].

### 4.2. Dietary Intake, Bacterial Composition, and Parkinson’s Disease

The comparison of the intake of nutrients between groups showed that dietary items like trans fatty acids seem to be taken up more in PD patients, and we also established a significant positive correlation involving the number of trans fatty acids and the severity of PD clinical manifestations, the years of illness and the Webster score (rs = 0.56, 0.44, 0.56). An increased uptake of trans fatty acids in the gut can result in alterations in the composition and biodiversity of the gut microbiota, induce inflammation, and alter the innate immunity of the intestinal tract [90]. Carbohydrates, on the other hand, as an important component of the diet, are one of the factors that affect the composition and metabolic function of the gut microbiota [91]. There was a decreased uptake of carbohydrates in the PD group, and there was a negative correlation between carbohydrate intake and PD severity, years of illness, and Webster score (rs = −0.63, −0.64, −0.62) in our cohort. The carbohydrates include sugars, starches, and dietary fibers [92]. Although long-term habits can determine the members of the microbiota, acute dietary changes, mostly in macronutrients and fiber, can significantly induce changes [93,94].

Even though our findings support the notion of an influence of decreased carbohydrate intake on PD progression, as opposed to the effect associated with the increased uptake of trans fatty acid, it is important to state that the evidence regarding the appropriate carbohydrate/fat ratio for PD is still inconclusive and conflicting [95]. For example, low-carbohydrate, high-fat diets are claimed to have positive effects on brain function [96]. Early evidence indicates that dietary manipulation may influence motor and cognitive symptoms in Parkinson’s disease. On one hand, a high-fat, high-protein diet may facilitate the passage of the dopamine precursors tyrosine and tryptophan into the brain and trigger an insulin-sensitive rise in brain dopamine [97,98]. However, the carbohydrate polymers that are not hydrolyzed by the endogenous enzymes and absorbed in the human small intestine [94] are instead metabolized by the gut bacteria in the large intestine. Through the fermentation of these fibers as an energy source, products like SCFAs and gases (H_2_, and CO_2_) are generated, which have been shown to benefit aspects of human health [99] like epithelial cell integrity, immune function, glucose homeostasis, lipid metabolism and regulation of appetite [100,101].

In our cohort, the PD patients showed a smaller intake of potassium compared with healthy controls, which, based on other studies, may be related to the impaired intestinal barrier and may alter the cellular signaling in the basal ganglia. Recently, a study of mice reported that a low potassium diet could increase intestinal permeability, which may result in bacterial translocation [102]. Additionally, changes in potassium could influence the regulation of K(+) channels and are important for cellular signaling in the basal ganglia; therefore, impairments in potassium could also have a relation with PD disease [103,104,105].

### 4.3. Metabolism of Aromatic Amino Acids Pathways Was Increased in Parkinson’s Disease Patients’ Microbiota

In our study, the functional abundance prediction analysis showed an increase in abundance of metabolic pathways in PD. In the first place we found aerobic respiration, which could be associated with aerobic facultative organisms in the gut. Bacteria such as the *Enterobacteriaceae* class are present in the gut at low levels, due to respiratory acceptor restriction (e.g., oxygen); however, under dysbiosis, the gut metabolism may change due to an increase in the oxygen levels in the gut, increasing the abundance of members of *Enterobacteriaceae* pathogens and generating inflammatory responses [106]. Other metabolic pathways, such as sucrose biosynthesis I (SUCSYN-PWY) and sucrose biosynthesis III (PWY-7347), in the PD cohort are involved in the generation of sucrose by glycerone phosphate and 3-phosphoglycerate, and the sucrose biosynthesis III pathway uses fructose-6-phosphate and UDP-α-d-glucose (an intermediate product of SUCSYN-PWY) to produce sucrose, uridine diphosphate, and phosphate [107].

Additionally, pathways related to aromatic compounds and catechol degradation were increased in abundance in PD patients—catechol degradation III (ortho-cleavage pathway, PWY-5417), aromatic compounds degradation via β-ketoadipate (PWY-5431), Catechol degradation I (PWY-5415) and catechol degradation to β-ketoadipate (CATECHOL-ORTHO-CLEAVAGE-PWY). To our knowledge, this is the first study that shows an association between these pathways and PD, although a study performed in the Chinese population found less prevalence of these metabolic pathways based on long-term diet quality [108].

Aromatic amino acid catabolism can generate a repertoire of aromatic by-products, some of which can be neurotransmitters and some of which can be harmful [109]. Bacterial species belonging to the *Clostridium* cluster XIVa, *Lactobacillus*, and *Lachnospiraceae* can metabolize aromatic amino acids like tyrosine to produce phenol and p-cresol [109]. These metabolites are associated with the loss of the structural integrity of intestinal epithelial cells and the consequently reduced stability of the gut epithelium [109]. Moreover, several species of the genus *Clostridium* are able to metabolize tyrosine and phenylalanine to yield phenolic metabolites, which can be used by other gut bacteria to produce p-cresol [110,111].

### 4.4. Personalized Metabolic Modeling of the Gut Microbiomes Revealed Parkinson’s Disease-Associated Microbial Metabolites 

The gut microbiota is part of human metabolism, due to a continuous interchange of substrates between the host and the microorganisms that are living in the gut. Metabolic modeling can show the functional and metabolic activities as a result of a change in the environment. We observed potential signals in the concentrations of indole, tryptophan, fructose, phenylacetic acid, 3-Methyl-2-oxovaleric Acid and N-Acetylneuraminic acid in PD.

Dietary tryptophan is the principal substrate for a diverse group of bacteria that are involved in the generation of indole derivatives in the gut (e.g., *Clostridium* spp., *Bacteroides*, and *Lactobacillus*) [112]. The healthspan benefits of indoles in organisms like *Caenorhabditis elegans* and others have been reported [113]. Indoles produced by the microbiota seem also to influence the age of onset and rate of progression of other neurodegenerative diseases, such as Alzheimer’s disease AD [114]. Moreover, indole and its derivatives are signaling molecules involved in the crosstalk with the host. Indole has been associated with intestinal immune homeostasis and barrier function by activating AhR and promoting the expression of IL-10 [115]. Additionally, these indole components influence the production of incretin through voltage-gated Ca^2+^, decreasing Adenosine triphosphate ATP regulation in L cells [116]. Thus, a diet rich in amino acids could promote the production of indole, and diets rich in sugar suppress its production and function in the gut [117].

In the diet from the healthy group, an increase in protein consumption was evident, although not significantly, and, therefore, a high consumption of amino acids are available for bacterial metabolism. This is reflected in the computational modeling, with an increase in the metabolism of aromatic amino acids, such as tryptophan and its derivatives, such as phenyl acetic acid and indole. The latter interacts with the intestinal epithelium, promoting the expression of anti-inflammatory cytokines and also the maintenance of tight-junction proteins [118]. Tryptophan metabolism and its modulation by the intestinal microbiota seem to play an important role in gut–brain communication; for example, it has been reported that serotonin synthesis, the kynurenine pathway, and microbial degradation pathways modulate physiology and enteric signaling regarding the generation of anti-inflammatory mediators and the availability of tryptophan, so that they can enter the brain [119,120]. For example, butyrate, which can enter the circulation, allows for the communication of the availability of tryptophan to the brain, inhibiting pathways degrading tryptophan to kynurenine, and, by this mechanism, increasing the generation of serotonin, in addition to its role in maintaining the gastrointestinal barrier with the compounds generated as indole [118]. According to this, tryptophan metabolism appears to play an important role in the microbiota–gut–brain axis.

Phenylacetic acid is one of the main products of the fermentation of aromatic amino acids, like phenylalanine produced by the phenylpropanoid pathway [110]. The main bacterial products are phenylacetic acid, phenylpropanoic acid, and benzoic acids [121]. The organisms involved in its production are species such as *Bacteroides*, *Eubacterium*, and *Clostridium* [110]. Phenylacetic acid is involved in the gut–liver axis and its concentration and increased levels of circulating BCAAs are associated with steatosis progression [122].

Fructose concentration was found to be increased in healthy phenotype simulations; this could suggest that fructose intake is simply higher in the control group. However, studies have shown that the increase in fructose is detrimental in excess. Sedentary habits and a diet high in fat and sugar, such as the Western diet, have been heavily associated, with high-fructose corn syrup, with obesity, diabetes, and others [123]. Fructose is an important nutrient that is metabolized in synergy with glucose. In the normal diet, it is present in fruits, and the liver is the major site of its metabolism after passing through the gut [124]. Fructose can modulate the phosphorylation of tyrosine pp185 on the insulin receptor; thus, insulin resistance and an increase in glucose are affected [125]. Additionally, high-fructose corn syrup can affect dopamine DA release, which is important in mesolimbic and nigrostriatal regions.

On the other hand, in the gut, excess dietary fructose consumption has been reported to be associated with a pro-colitis effect that can be explained by changes in the composition and metabolic function of gut microbiota [126]. However, the source of fructose seems to have an impact on the composition of the microbiome. A fructose fruit diet increases the abundance of the phylum *Firmicutes* (*Faecalibacterium*, *Anaerostipes*, and *Erysipelatoclostridium*) and decreases the abundance of the phylum Bacteroidetes (*Parabacteroides*). Alternatively, diets rich in high-fructose corn syrup reduced the abundance of *Firmicutes* and the genus *Ruminococcus* and increased the abundance of *Bacteroidetes*. The increased abundance of *Bacteroidetes* was correlated with plasma cholesterol and LDL levels [127]. This suggests that diets containing fructose from fruit sources modulate the composition of the gut microbiota in a beneficial way and that a high fructose intake, via high-fructose corn syrup, causes a reduction in beneficial butyrate-producing bacteria, promoting alterations in the lipid metabolism and gut barrier homeostasis [127].

Myristic acid, which was increased in the Parkinson’s disease phenotype, is found in dairy dietary sources like butter, coconut, and palm oils [128]. It has been associated with cardiovascular diseases and with increasing LDL and total cholesterol, in diets beyond 4% of total energy [129]. Additionally, this saturated fatty acid induces ceramide synthesis via CerS5 and, consequently, increases autophagy in cardiomyocytes [130]. In mice and primary mouse hepatocytes, myristic acid, together with palmitic acid, increased the production of ceramide and cholesterol levels, inflammation, and fibrosis. These fatty acids are involved in de novo ceramide production and Parkinson’s disease [131].

Additionally, N-Acetylneuraminic acid and 3-Methyl-2-oxovaleric acid concentration were found to be increased in healthy phenotype simulations. N-Acetylneuraminic acid is an indispensable part of sialic acid [132]. A major source of sialic acid in the intestine are mucins, which are the main structural components of the mucous layers lining the gastrointestinal tract. In addition, they may also come from dietary sources, such as milk and meat. These components may have an important role in the intestinal bacterial composition, as they possess diverse pathways of release, metabolization, and transport of derivatives, allowing bacterial crosstalk relationships to be established [133]. Thus, alterations in the homeostatic levels of sialic acid in the intestine have been associated with infections and inflammation in preclinical models [134], but the underlying mechanisms have not yet been discovered. A better understanding of the sialic acid derivatives metabolized by intestinal microbes and their role in signaling would allow us to propose new biomarkers and therapeutic targets.

3-Methyl-2-oxovaleric acid, a keto acid that is a subclass of organic acids [135], significantly increased in response to fruit intake-based keto acids. Keto acids are formed from the enzymatic deamination of amino acids, carried out in part by gut bacteria [136]. On the other hand, chronically high levels of 3-Methyl-2-oxovaleric acid are associated with maple syrup urine disease. This is a metabolic disorder caused by a deficiency of the branched-chain alpha-keto acid dehydrogenase complex, leading to a buildup of the branched-chain amino acids and their toxic by-products (ketoacids) in the blood and urine. In maple syrup urine disease, the brain concentration of branched-chain keto acids can increase and cause neurological damage [137,138].

## 5. Conclusions

To our knowledge, this is the first study that characterizes the gut bacterial composition of PD patients and healthy controls from a cohort in the Colombian population and in Latin America. We were able to identify a group of bacterial families and genera that are associated with PD, in terms of their abundance, showing that our results are complementary and also comparable, to some extent, with studies from other populations around the world.

Our results also reveal some differences in microbiome composition between the Latin American population and former studies. Therefore we hypothesize that there are no “global” and “generalized” microbial compositions related to PD for all humans, but rather they appear to be specific in each population. This emphasizes the importance of the characterization of specific populations, the Colombian one in our case, to better respond to local challenges.

According to our analysis, many of the bacterial families and genera associated with the zOTUS that we have identified as significantly associated with PD status have shown an association with gut inflammation in prior studies. We were also able to identify an induction of aromatic amino acid metabolism in PD patients that could be modulated by its consumption in the diet and/or levodopa medication, although more statistical power to identify confounders in our cohort is necessary.

Finally, we were also able to create personalized models of the gut community for individual patients, which adds to the growing global efforts in the field of personalized medicine. This also allowed us to identify potential metabolites related to PD that could be used as potential biological markers of disease. This highlights the importance of holistic approaches and shows how systems biology could be used in the study of neurodegenerative diseases. It is also important to stress the necessity of improving statistical and computational tools, to integrate data from diverse omics layers, to provide a better understanding of the underlying biology and microbiome–host interactions. As a consequence, this opens venues to use computational modeling as a central component of personalized therapeutic approaches.

## Figures and Tables

**Table 1:** Demographic and clinical parameters comparison between Parkinson’s patients and control subjects.

Variables	Control Subjects (% (n)/Median [IQR])	Parkinson’s Patients (% (n)/Median [IQR])	*p*. Adjusted	Test
Demographics				
Sex	30 (15)	28 (14)	1	Fisher
Smoke	16 (8)	30 (15)	0.526	Fisher
Alcohol	10 (5)	34 (17)	**0.012**	Fisher
Weight (kg)	68.7 [17.4]	62.2 [17.2]	0.265	Wilcoxon
Medication				
Levodopa/carbidopa	0	36 (18)	**5.33 × 10^−5^**	Fisher
Pramipexole	0	12 (6)	0.156	Fisher
Phenobarbital	0	2 (1)	1	Fisher
Rotigotine	0	6 (3)	1	Fisher
Mirapex	0	4 (2)	1	Fisher
Metformin	0	4 (2)	1	Fisher
Disease related variables				
Stools per week	7 [4]	7 [3]	0.677	Wilcoxon
Bristol scale	4 [1]	3 [1]	**0.001**	Wilcoxon
Webster scale	0	11 [5]	**9.413 × 10^−8^**	Wilcoxon
Calf perimeter	37.1 [3.8]	35.5 [3.5]	0.013	Wilcoxon

Statistically significant *p*-values are marked in bold font. IQR: interquartile Range, n: number of participants.

**Table 2:** Analysis of similarities (Anosim) and permuted multivariate analysis of variance (Adonis) of zOTUs between PD patients and controls.

Distance	Adonis_R	Adonis_*p*-Value	Anosim_R	Anosim_*p*-Value
Braycurtis	0.026	0.1224	0.019	0.1944
Uwunifrac	0.033	**0.0083**	0.059	**0.016**
Wunifrac	0.039	0.0924	0.083	**0.0052**

Statistically significant *p*-values are marked in bold font.

## Data Availability

All datasets generated for this study are included in the manuscript and/or the Supplementary Files. All raw sequence data generated in this study have been deposited in NCBI under BioProject accession number PRJNA975118, https://www.ncbi.nlm.nih.gov/bioproject/PRJNA975118 (accessed on 1 December 2023). Registration date: 15 June 2023. Code available in Github repository: https://github.com/ljforeror/Parkinson_Microbiome (accessed on 1 December 2023).

## Supplementary Materials

The following supporting information can be downloaded at: https://www.mdpi.com/article/10.3390/microorganisms12020325/s1. Figure S1: heat map representing the correlation analysis performed between nutritional information and the main zOTUS. Figure S2: representation of the top 20 dietary metabolites acting as contributors to the variation in PD patients and controls in Dimension 1. The main taxpayers are above the threshold indicated as a red line. Figure S3: representation of the top 20 dietary metabolites acting as contributors to the variation in PD patients and controls in Dimension 2. Figure S4: rarefaction curve based on the number of zOTUs found in each of the samples at different sequencing depths. After the depth of rarefaction. Figure S5: heatmap of the relative abundance of phylum in patients with PD and healthy controls. Figure S6: NMDS representation of the relationship of bacterial composition in PD patients and control groups. Figure S7: the bar graph represents the concentration of short-chain fatty acids in the metabolic reconstruction simulations of PD patients and controls. Figure S8: differential metabolite-consuming bacteria *in silico* between phenotypes. Figure S9: *in silico* short chain fatty acid-consuming bacteria between phenotypes. Figure S10: *in silico* short chain fatty acid-producing bacteria between phenotypes. Table S1: nutritional assessment and percentage of the participants. Table S2: nutritional assessment by groups. Table S3: Spearman correlation values between clinical data and metabolites from the diet and *p*-values. Table S4: Spearman correlation values between zOTUs and metabolites from the diet and *p*-values. Table S5: Wilcoxon test diet metabolites, non-corrected. Table S6: Wilcoxon test diet metabolites, corrected. Table S7: Adonis test for possible confounders. Table S8: zOTUs with significant abundance < 0.1. Table S9: zOTUs with significant abundance > 0.1. Table S10: significant differential families. Table S11: significant differential phylum. Table S12: Deseq2 analysis of MetaCyc pathways (PICRUSt 2). Table S13: individualized dietary uptake *in silico* models. Table S14: GLM analysis from metabolite concentration in the *in silico* models. Table S15: Wilcoxon and Kruskal–Wallis test of exchange fluxes in the *in silico* models. Table S16: bacteria exchange reactions from SCFAs from *in silico* models. Table S17: bacteria exchange reactions from metabolites with differences in concentration from *in silico* models.
