# Supplementary material for: Changes in Bacterial Gut Composition in Parkinson’s Disease and Their Metabolic Contribution to Disease Development: A Gut Community Reconstruction Approach
Source: Microorganisms. 2024 Feb 4;12(2):325. doi: 10.3390/microorganisms12020325 (PMC10893096; doi:10.3390/microorganisms12020325)
Supplement: Supplementary file 1 [file microorganisms-12-00325-s001.zip › Supplementary-microorganisms-2797537/Supplementary/Figures/Supplementary_Figures.docx]

**Supplemental material for:**

**Johanna Forero-Rodríguez ^1,2^, Johannes Zimmermann ^2^, Jan Taubenheim ^2^, Natalia Arias-Rodríguez ^1^, Juan David Caicedo-Narvaez ^1,6^, Lena Best ^2^, Cindy V. Mendieta ^3,4^, Julieth López-Castiblanco ^1^, Laura Alejandra Gómez-Muñoz ^6,7^ , Janneth Gonzalez-Santos ^5^, Humberto Arboleda ^7^, William Fernandez ^6,7^, Christoph Kaleta ^2,#^, Andrés Pinzón ^1,#^.**

|  |
| --- |

^1^ Bioinformatics and Systems biology Research Group, Genetic Institute, Universidad Nacional de Colombia, Bogotá, Colombia.

^2^ Research Group Medical Systems Biology, Institute of Experimental Medicine, Christian-Albrechts-Universität zu Kiel, Germany.

^3^ PhD Program in Clinical Epidemiology, Department of Clinical Epidemiology and Biostatistics, Faculty of Medicine, Pontificia Universidad Javeriana, Bogotá, Colombia.

^4^ Department of Nutrition and Biochemistry, Pontificia Universidad Javeriana, Bogotá, Colombia.

^5^ Structural Biochemistry and Bioinformatics Laboratory, Pontificia Universidad Javeriana, Bogotá, Colombia.

^6^ Neurosciences Research Group, Genetic Institute, Universidad Nacional de Colombia, Bogotá, Colombia.

^7^ Cell Death Research Groups, Medical School and Genetic Institute, Universidad Nacional de Colombia, Bogotá, Colombia.

^#^ These authors jointly supervised this work

***** Correspondence: Andrés Pinzón ^1, #^, Research Group Bioinformatics and Systems Biology, Instituto de Genética, Universidad Nacional de Colombia, Bogotá, Colombia. Email: ampinzonv@unal.edu.co and Christoph Kaleta ^2, #^ Research Group Medical Systems Biology, Institute of Experimental Medicine, Christian-Albrechts-Universität zu Kiel, Germany. Email: c.kaleta@iem.uni-kiel.de.

#### **Correlation analysis between dietary variables and significative differential abundant zOTUs**

To identify if there are dietary contributors to the presence of the significant differential zOTUS in the context of PD Additionally, we assessed correlations between the 65 dietary variables and the six differentially abundant zOTUS. 37 nutrients had non-significant correlations after correction regarding the bacterial families, the remaining nutrients did not show statistical significance. The *Family: Lachnospiraceae Genus: Clostridium_XlVa*, correlates with trans fatty acids and fiber rather than protein. *Genus: Intestinibacter*, correlates with vitamin K, vitamin D, and sodium and has a negative correlation with ascorbic acid. Family: *Lactobacillaceae*, Genus: *Lactobacillus* is positively correlated with cholesterol and carbohydrate intakes. *Genus: Streptococcus (zOTU 31)* correlates with diets rich in caffeine, vitamin A, and vitamin E. The *Genus: Streptococcus* , correlates with cholesterol and carbohydrates. Finally, the *Genus: Akkermansia ,* correlates with rich in amino acids, carbohydrates, fiber, potassium, magnesium, stearic acid, total fat, copper, and vitamins (vitamin A and thiamine) (Supplementary Table 4, Supplementary Figure 1). These findings show that although there are no significant values, could indicate possible dietary compounds that may influence the bacterial gut composition.


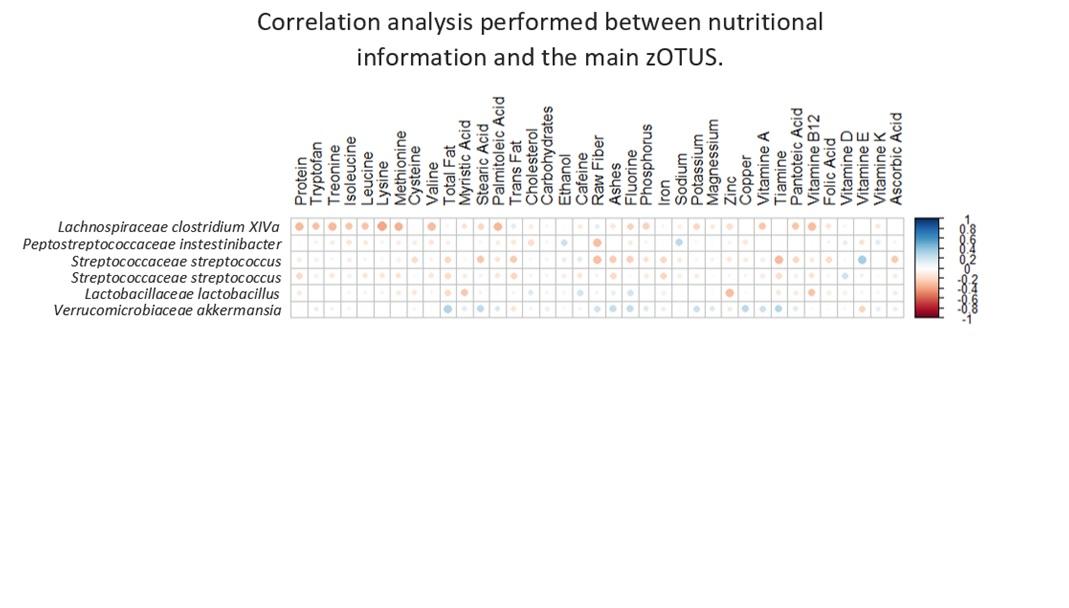


**Supplementary Figure 1.** Heat map representing the correlation analysis performed between nutritional information and the main zOTUS.

#####
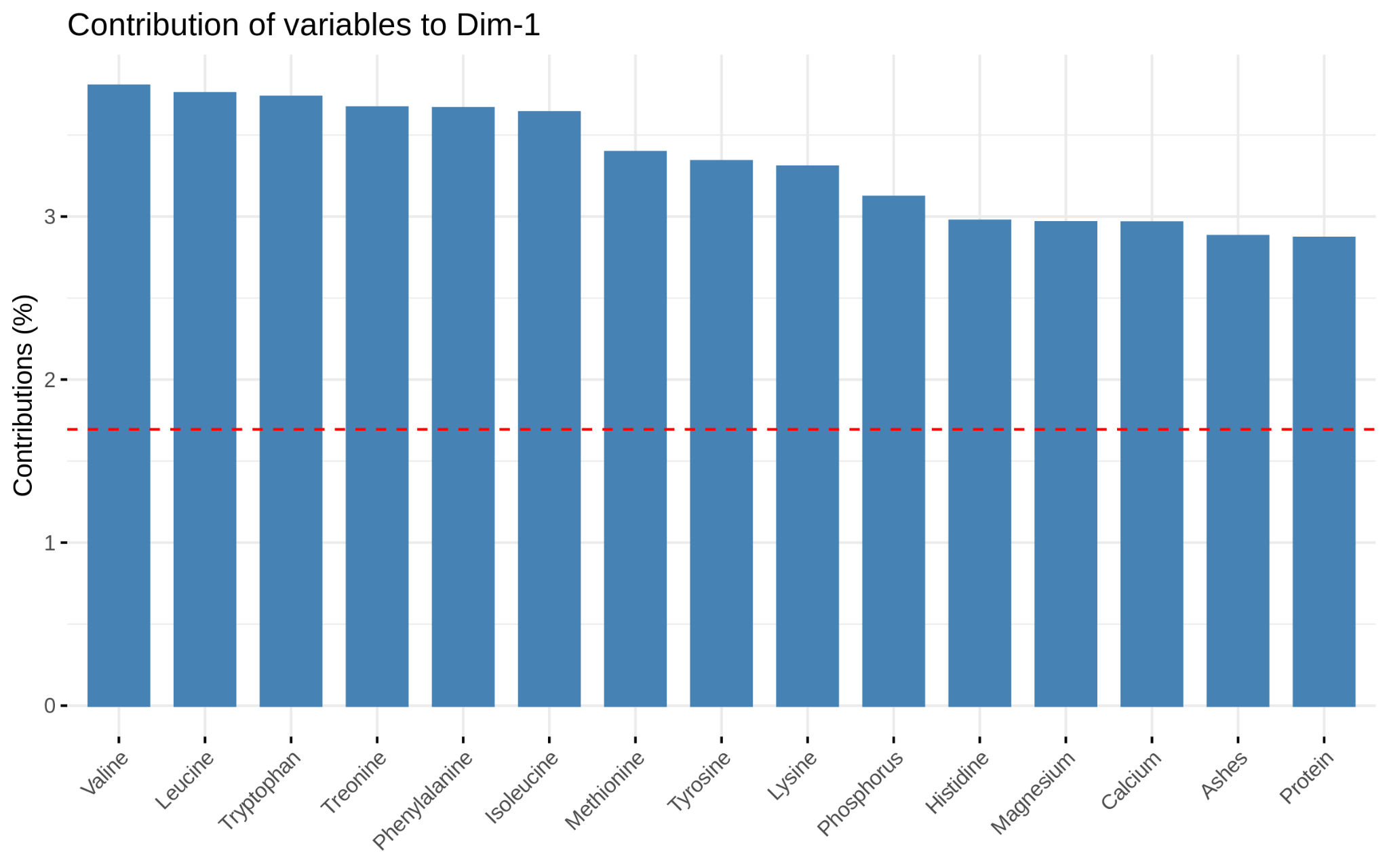


##### **Supplementary Figure 2.** Representation of the top 20 dietary metabolites acting as contributors to the variation of PD patients and controls in Dimension 1. The main taxpayers are above the threshold indicated as a red line.

#####

#####
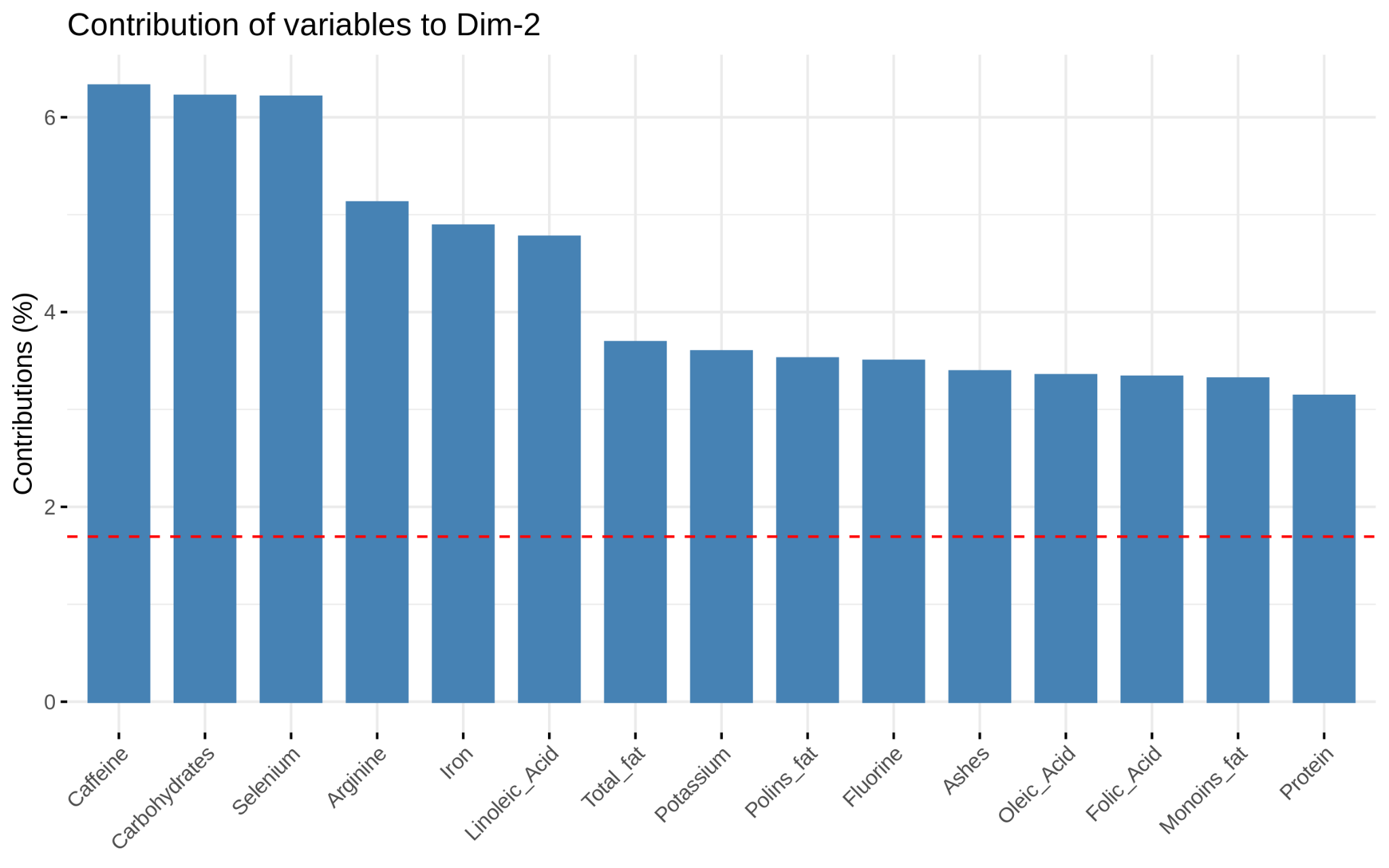


##### **Supplementary Figure 3**. Representation of the top 20 dietary metabolites, acting as contributors to the variation in PD patients and controls in Dimension 2.

##### The main taxpayers are above the threshold indicated as a red line.


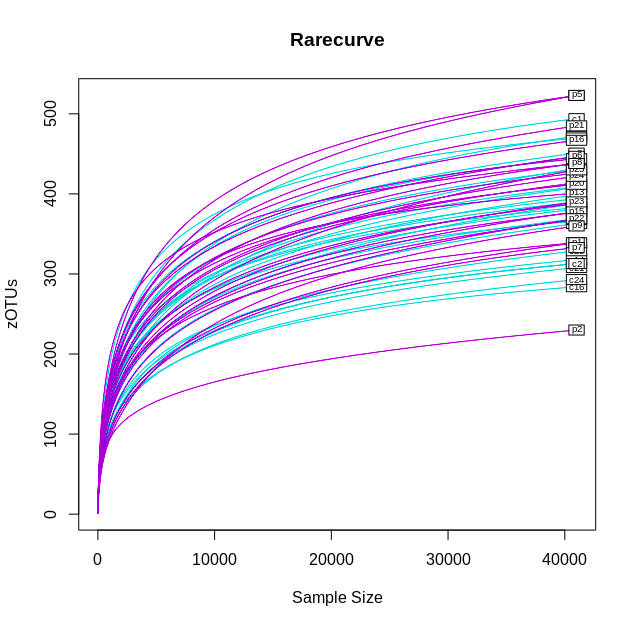
**Supplementary Figure 4.** Rarefaction curve based on the number of zOTUs found in each of the samples at different sequencing depths. After the depth of rarefaction.

#####

#####
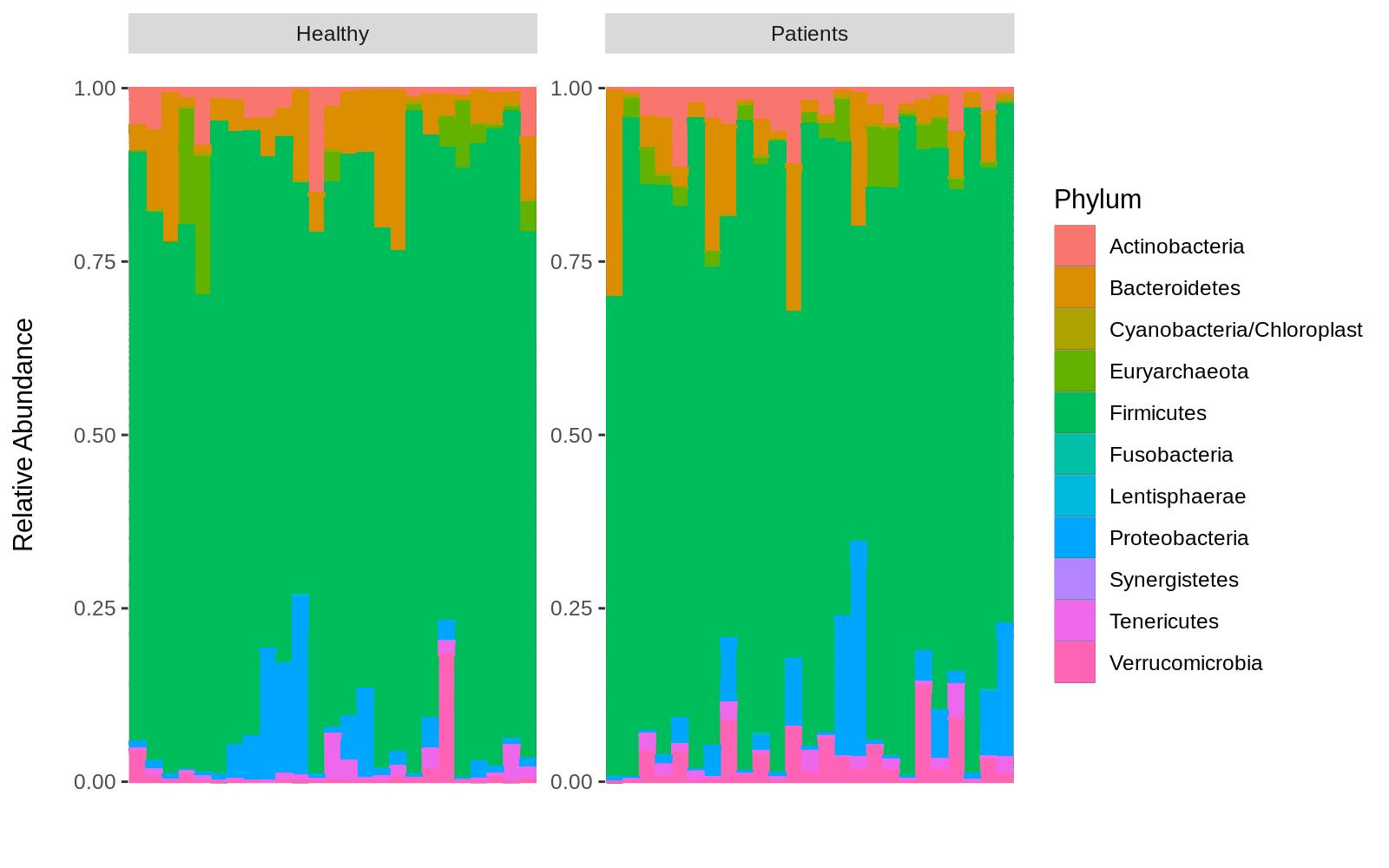


**Supplementary Figura 5.** Heatmap of the relative abundance of Phylum in patients with PD and healthy controls. Each group is composed of 25 participants.


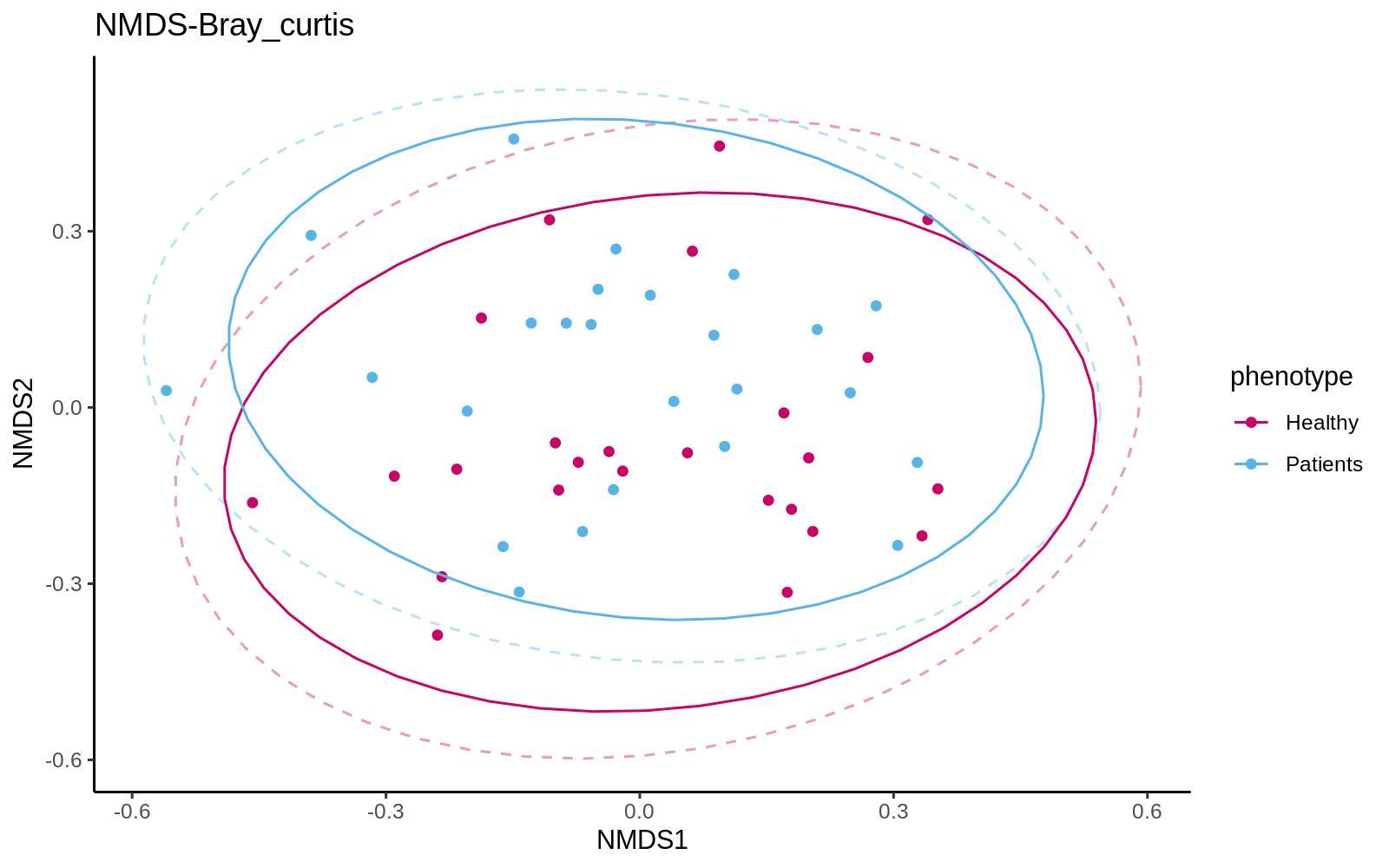


**Supplementary Figure 6.** NMDS representation of the relationship of bacterial composition in PD patients and control groups. The composition of the samples was compared using the Bray Curtis distances with the normalized abundances.

#####
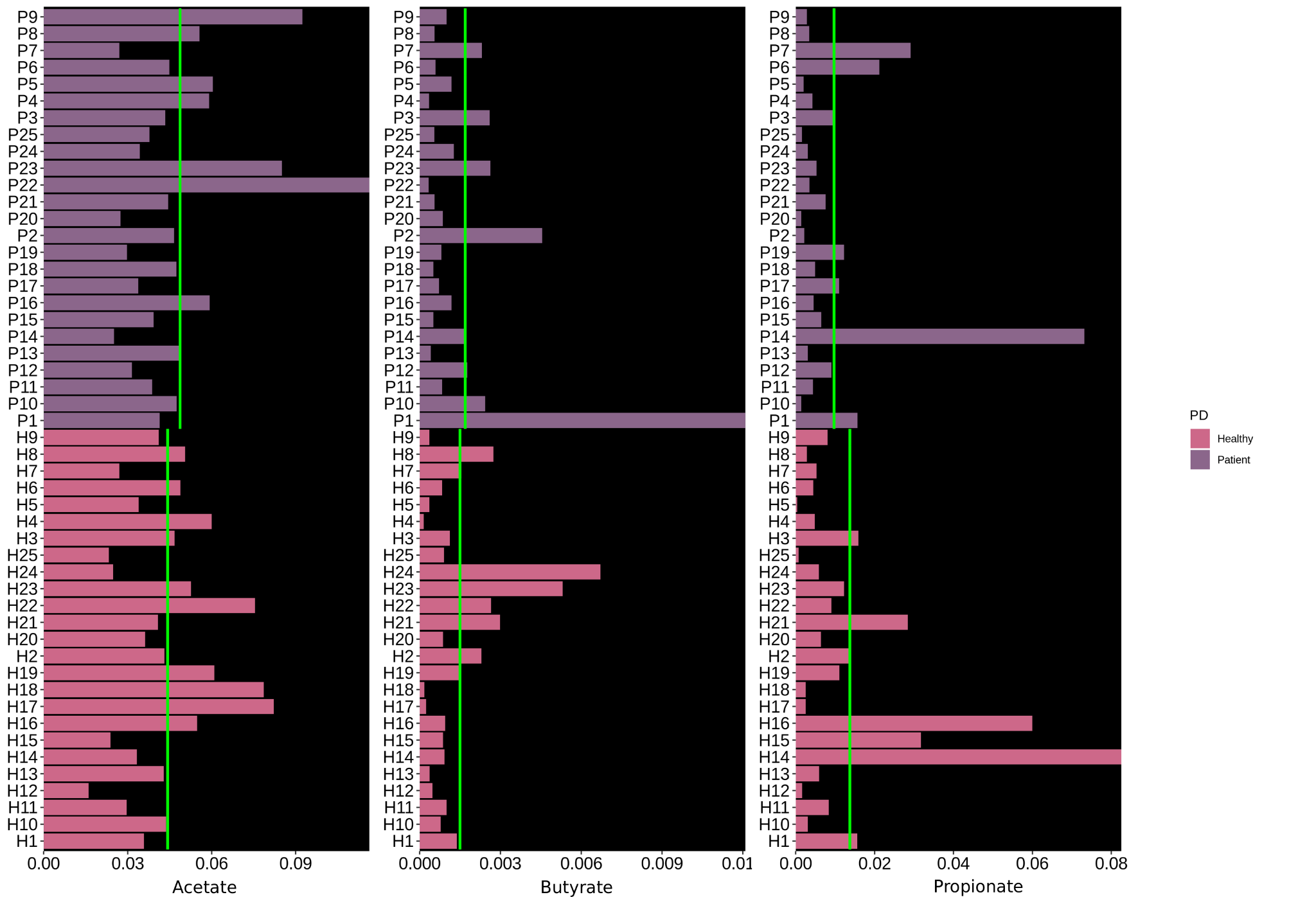


**Supplementary Figure 7.** The bar graph represents the concentration of short-chain fatty acids in the metabolic reconstruction simulations of PD patients and controls. mM concentration of the 12H simulation in each participant belonging to each group, PD patients (blackberry) and controls (pink).


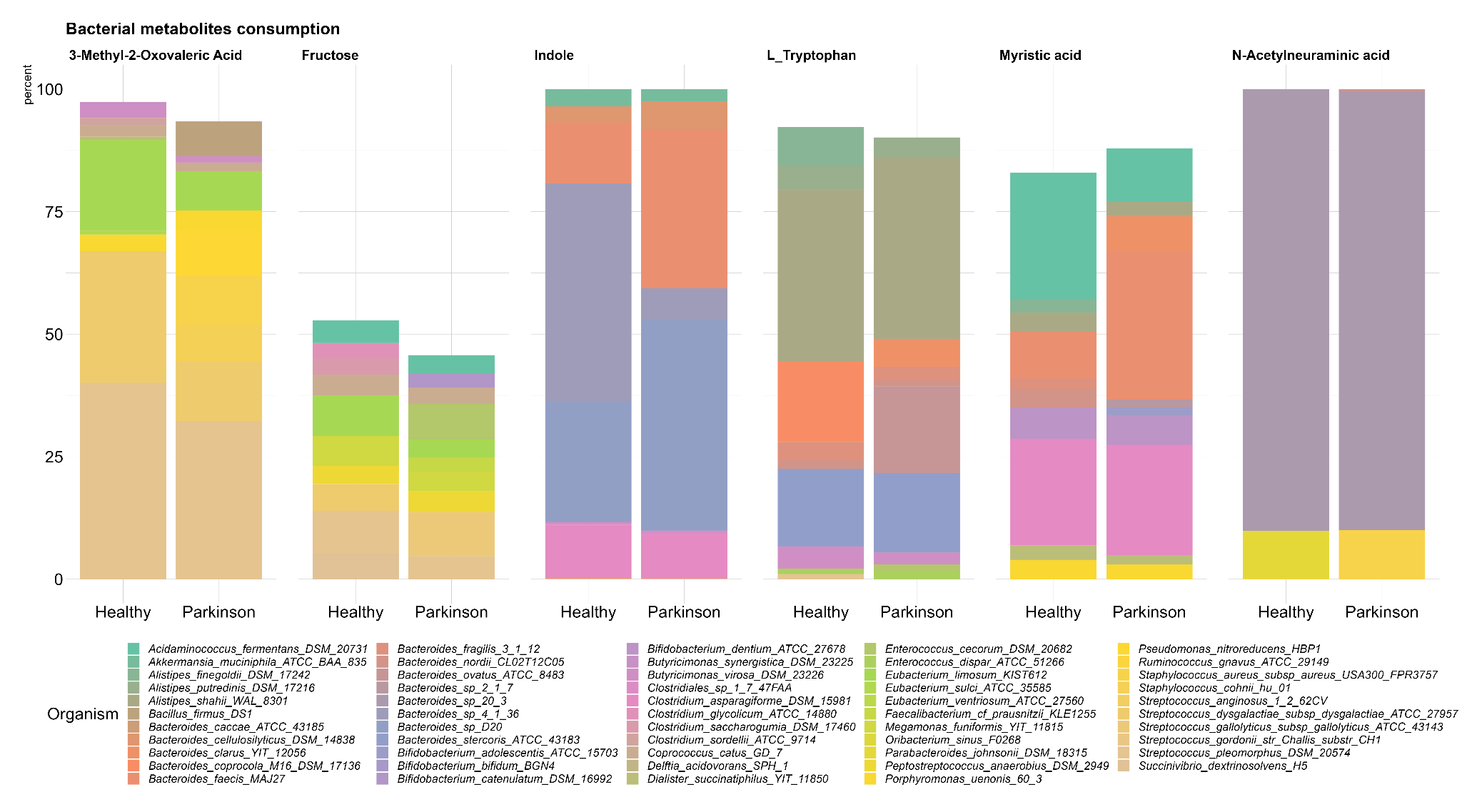


**Supplementary Figure 8.** Differential metabolite-consuming bacteria in silico between phenotypes. Top 10 of the largest consumers of 3-Methyl-2-Oxovaleric Acid, Myristic Acid, Indole, Fructose, Phenylacetic Acid and N-acetylneuraminic acid. The percentage of consumption for each organism is indicated for the first ten consumers.


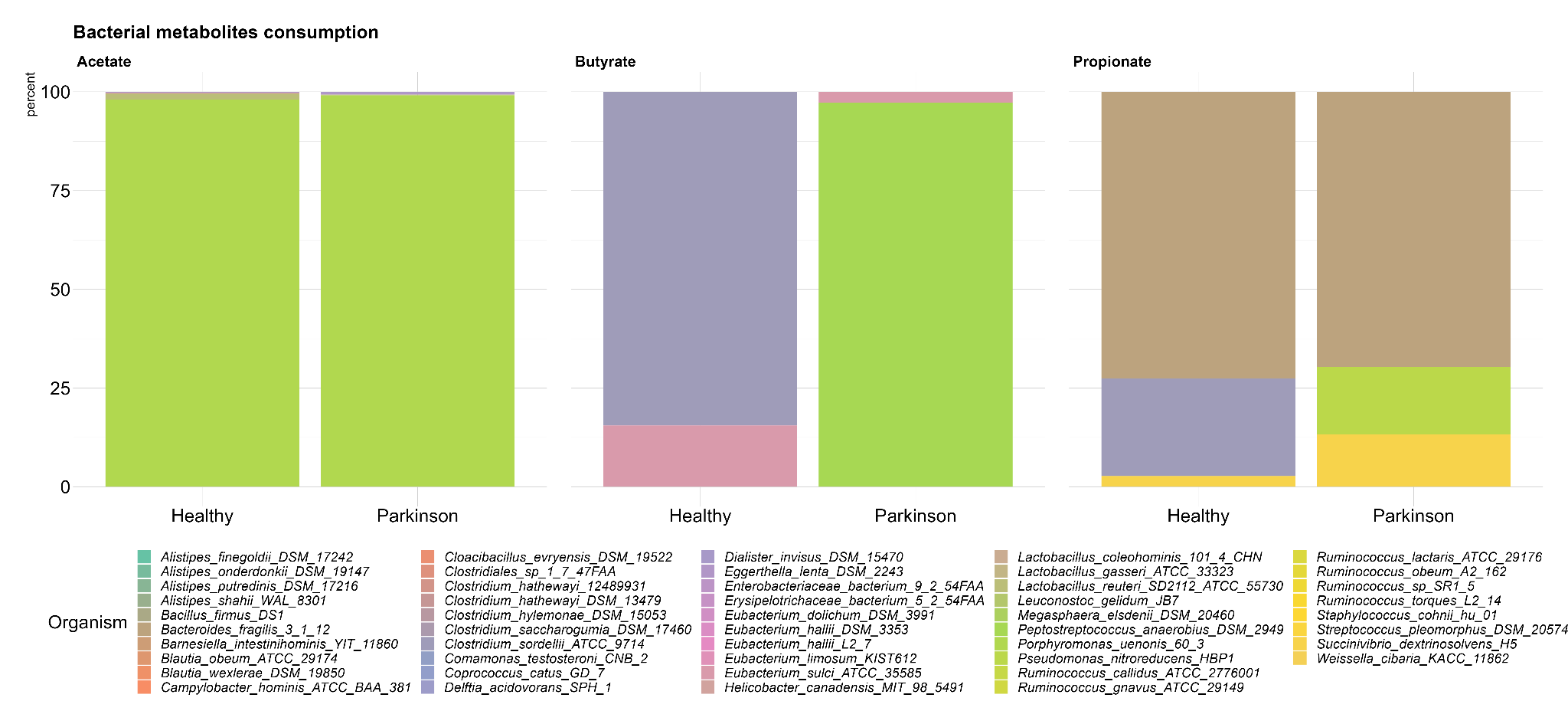


**Supplementary Figure 9.** *In silico* short-chain fatty acid-consuming bacteria between phenotypes. The percentage of consumption and production for each organism is indicated for the first ten organisms.


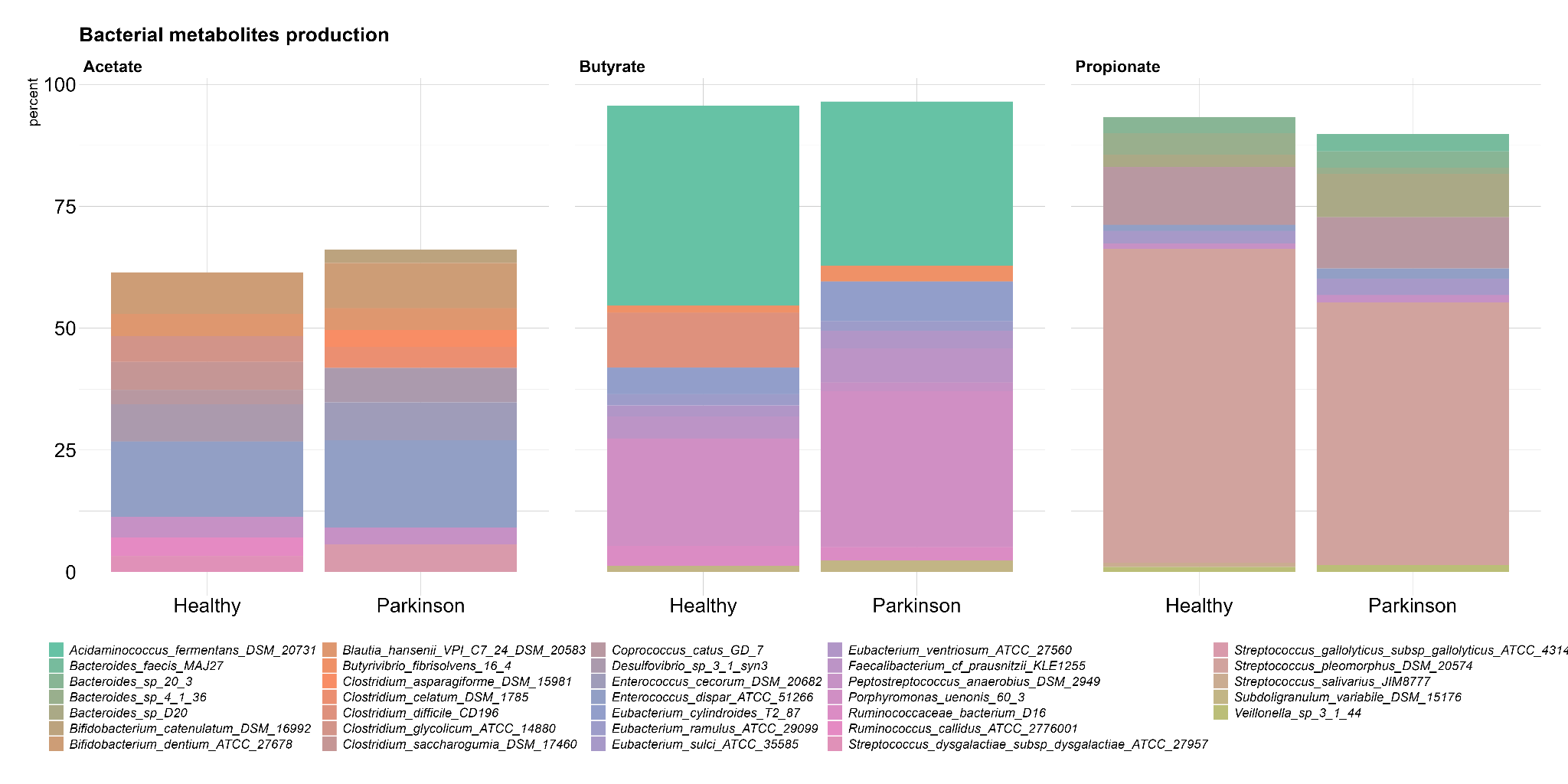


**Supplementary Figure 10.** *In silico* short-chain fatty acid-producing bacteria between phenotypes. The percentage of consumption and production for each organism is indicated for the first ten organisms.
